# Advances in Membrane Distillation Module Configurations

**DOI:** 10.3390/membranes12010081

**Published:** 2022-01-12

**Authors:** Lijo Francis, Farah Ejaz Ahmed, Nidal Hilal

**Affiliations:** NYUAD Water Research Center, New York University-Abu Dhabi, Abu Dhabi P.O. Box 129188, United Arab Emirates; lf2426@nyu.edu (L.F.); farah.ahmed@nyu.edu (F.E.A.)

**Keywords:** advanced membrane distillation, membrane modules, pilot scale studies, water treatment, brine treatment, desalination

## Abstract

Membrane Distillation (MD) is a membrane-based, temperature-driven water reclamation process. While research emphasis has been largely on membrane design, upscaling of MD has prompted advancements in energy-efficient module design and configurations. Apart from the four conventional configurations, researchers have come up with novel MD membrane module designs and configurations to improve thermal efficiency. While membrane design has been the focus of many studies, development of appropriate system configurations for optimal energy efficiency for each application has received considerable attention, and is a critical aspect in advancing MD configurations. This review assesses advancements in modified and novel MD configurations design with emphasis on the effects of upscaling and pilot scale studies. Improved MD configurations discussed in this review are the material gap MD, conductive gap MD, permeate gap MD, vacuum-enhanced AGMD/DCMD, submerged MD, flashed-feed MD, dead-end MD, and vacuum-enhanced multi-effect MD. All of these modified MD configurations are designed either to reduce the heat loss by mitigating the temperature polarization or to improve the mass transfer and permeate flux. Vacuum-enhanced MD processes and MD process with non-contact feed solution show promise at the lab-scale and must be further investigated. Hollow fiber membrane-based pilot scale modules have not yet been sufficiently explored. In addition, comparison of various configurations is prevented by a lack of standardized testing conditions. We also reflect on recent pilot scale studies, ongoing hurdles in commercialization, and niche applications of the MD process.

## 1. Introduction

Water scarcity has emerged as one of the greatest global environmental challenges of the 21st century, owing to climate change, rapid urbanization, and unprecedented population growth. In 2020 alone, two billion people, or 26% of the world’s population, lacked safely managed drinking water [1]. In urban areas, not only is there an increased water demand for human consumption, but the discharge of industrial and domestic wastes has also severely deteriorated the water quality of existing resources [2].

Consequently, the last few decades have witnessed heightened interest in the search of cost-effective technologies for providing freshwater to reduce water stress. Desalination and wastewater treatment allow us to obtain freshwater from saline and/or contaminated water resources with varying levels of salinity, which in turn allows us to treat and reuse wastewater, as well as tap into renewable and abundant resources such as seawater. Today, global desalination operating capacity amounts to over 78 million m^3^ per day across 183 countries [3]. Desalination technologies are typically categorized as membrane or thermal. While thermal distillation systems such as multi-stage flash (MSF) and multi-effect distillation (MED) have long been used to extract freshwater through desalination, their share in the desalination market has been surpassed by pressure-driven membrane processes; notably, reverse osmosis (RO) desalination. Membrane-based systems offer the advantage of lower space requirements and modular system design. In comparison to thermal desalination, RO is capable of desalinating at lower specific energy consumption [4,5] as it does not rely on phase change. However, conventional thermal separation processes still hold certain advantages as they are less susceptible to fouling [6], and less sensitive to feed salinity [7,8] with high separation efficiencies even for hypersaline feed solutions. Low-temperature distillation systems are promising when low-grade waste heat or solar heat is readily available [9,10,11], as is the case in several countries in/around the Arabian Gulf. Furthermore, RO is limited to the treatment of low to moderately saline solutions, and is not energetically feasible for the treatment of hypersaline feed solutions, such as desalination reject brine and produced water from oil and gas production, which may have total dissolved solids (TDS) of up to 200,000 ppm [12,13]. Thermal processes are more suited to such hypersaline solutions, as well as zero liquid discharge and recovery of valuable compounds from aqueous streams.

Membrane distillation (MD) is an emerging separation process that combines the compactness and modularity of membrane systems with distillation driven by low-temperature heating [14]. As a thermally driven process, MD is suitable for treating a wide range of feed types and can be applied to seawater desalination, brackish water desalination, and brine treatment. It is also applied in the food industry for the concentration of dairy streams [15,16,17], and in industrial processes for the removal of volatile organics [18,19,20,21,22].

**Figure 1:**
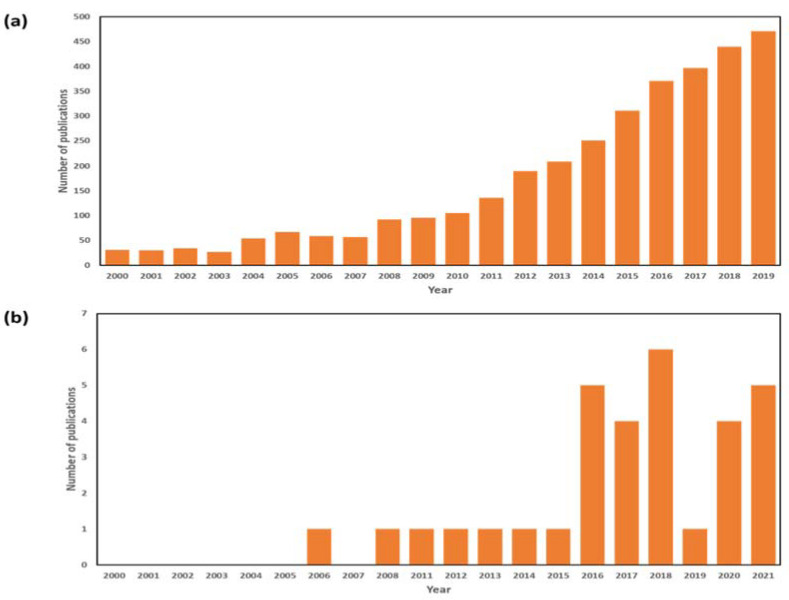
Number of publications on (**a**) ‘membrane distillation’, and (**b**) pilot scale MD studies between 2000 and 2020 (Scopus).

Figure 1 shows the number of publications on ‘membrane distillation’, as well as those focusing on pilot scale studies (Figure 1b). While pilot scale MD systems have not quite garnered the same attention as research in MD materials and configurations, a sharp rise in publications can be seen between 2016 and the preceding years. A shift in research direction from membrane materials to module and configuration design for upscaling has enabled MD to move closer to commercialization. While membrane development remains an important component [23,24,25] in overcoming challenges pertaining to MD, system configuration, which includes feed channel designs and heating methods, are equally essential aspects [26] in advancing MD technology.

To date, several reviews have been published on MD development [27,28,29,30,31,32]. Some have addressed advances in fouling, scaling, and wetting in MD systems, as it is cited as one of the most significant challenges in MD [33]. There has been extensive interest in the modeling of mass transport and fouling and wetting behavior in MD, for which the reader is referred to other reviews [28,34,35,36]. In light of the emphasis on membrane development in the past two decades, a few reviews have addressed membrane materials and fabrication methods, as well as techno-economic analysis of different membrane systems [37,38,39,40,41,42]. Wang and Chung briefly addressed MD configurations in their review which included membrane development, system design, and applications for MD [43]. However, there is no review to date that focuses entirely on MD module configurations. With growing developments in both novel and modified configurations for MD systems, and the status of MD as an emerging desalination technology, it is imperative to review advances in the area to guide future work in energy-efficient MD systems. This review assesses the role of new and modified MD system configurations in the route to commercialization for desalination and wastewater treatment as well as other niche applications. This review will also explore the gap between academic and industrial developments in MD systems to identify areas that need attention to further boost MD toward full-scale implementation.

### 1.1. Operating Principle

**Figure 2:**
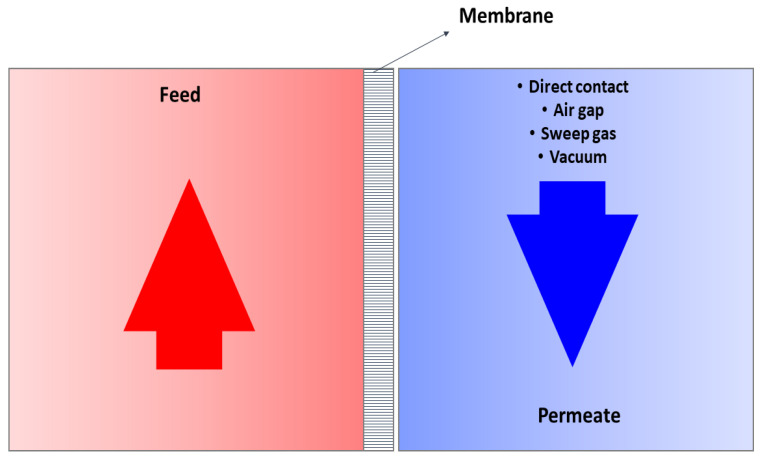
Schematic of the MD process.

MD is a thermally driven membrane separation process in which a thermal gradient across the membrane drives separation. A hydrophobic porous membrane is in direct contact with a heated feed solution and the vapor pressure difference caused by the temperature gradient vaporization at the liquid-vapor interface on the feed side [44,45]; formed vapor then passes through the membrane pores and condenses at on the permeate side [46]. The greater the temperature difference between both sides of the membrane, the greater the driving force and thus, greater the mass flux [47]. Various studies were carried out using different spacers and multi staging the different MD systems for increasing the efficiency of permeate production [48,49]. MD systems are operated at low temperatures <80 °C [50] and at or below atmospheric pressure [51,52,53]. A hydrophobic membrane ensures that only volatile vapor passes through the membrane, and the liquid feed is not penetrated through the pores into the permeate [46,54]. Figure 2 shows the schematic representation of the general MD process.

All MD configurations suffer from common challenges. First, 20–40% of energy supplied by the hot feed is lost by conduction through the membrane [27] The temperatures at the membrane surface are thus different from those of the bulk solutions. This gives rise to the formation of a thermal boundary layer at the membrane surface (on both feed and permeate). This phenomenon, known as temperature polarization (TP), is a limiting factor of mass transport efficiency [45,55].

Design considerations should aim to improve energy efficiency and take TP into account without hindering mass transport. Another commonly cited challenge for MD is wetting, which refers to penetration of the membrane pores by liquid feed. Wetting leads to poor permeate quality and can also lead to membrane fouling.

### 1.2. Conventional Configurations

There are four common MD configurations, each of which differs in the method of water collection or condensation.

#### 1.2.1. Direct Contact Membrane Distillation (DCMD)

In DCMD, the permeate side of the membrane is in direct contact with a pure water stream [56]. Due to its simplicity, DCMD is the most commonly used in lab-scale MD studies. However, high conductive losses due to contact of the membrane with both streams result in low energy efficiency; as a result, DCMD has found little use in pilot scale MD systems [57]. Nevertheless, more than 60% of lab-scale studies are carried out using this configuration [34]

Apart from the use of solar energy and low-grade waste heat, Ullah et al. emphasized the importance of module redesign and heat recovery for energy-efficient DCMD [58]. Another review of DCMD acknowledged the low energy efficiency of DCMD systems and suggested hybridization of DCMD with other processes [31]. Researchers have also reviewed the use of DCMD for textile wastewater treatment [59] and recovery of value-added compounds [60].

#### 1.2.2. Air Gap Membrane Distillation (AGMD)

In AGMD, an air gap separates the membrane from a cool condenser surface. The low conductivity of air limits conductive heat losses through the membrane. Mass flux in AGMD systems depends strongly on the width of the air gap. While the presence of an air gap reduces conductive heat loss, it also provides mass transfer resistance to vapor flow before reaching the condenser surface [61,62]. To operate at high flux, the air gap width must therefore be optimized for each system. Due to enhanced energy efficiency compared to DCMD, AGMD and VMD, are the preferred configurations for pilot scale MD systems, as shown in Section 3.

#### 1.2.3. Sweeping Gas Membrane Distillation (SGMD)

In SGMD, an inert gas sweeps the vapor in the permeate channel; the vapor is then condensed outside the membrane module. Similar to AGMD, an insulating gas barrier lowers conductive heat loss compared to DCMD, but the sweeping motion of the gas acts to improve mass transfer in the permeate channel [27]. SGMD is the least used MD configuration due to the need for an external condenser to collect the permeate, and a more complicated system design [34]. Said et al. provide a detailed review in which they explore the effect of gas temperature and flow rate on SGMD performance [63]. A positive correlation between gas flow rate and mass flux is observed due to increased Reynold’s number and reduction of hydrodynamic boundary layer thickness. The effect of gas temperature on mass flux however depends on the length of the membrane module.

#### 1.2.4. Vacuum Membrane Distillation (VMD)

VMD is an MD configuration in which vapor is extracted by applying a vacuum pressure of 5–10 kPa to the permeate side of the membrane [64,65]. Similar to SGMD, the vapor is recovered outside the module via an external condenser. Vacuum on the permeate side increases the vapor pressure gradient across the membrane, and thus results in increased flux. Although increasing vacuum pressure would result in higher permeate flux, exceeding the liquid entry pressure (LEP) of the membrane may result in membrane wetting and performance deterioration [66]. For deeper insight into the status of VMD research, the reader is referred to other reviews [29,32,67].

Table 1 shows the benefits and challenges associated with each of the conventional configurations.

Studies comparing MD configurations under similar conditions are surprisingly uncommon, mostly due to a lack of standardized testing conditions. van der Bruggen’s group investigated the effect of MD configuration on energy efficiency and flux [70] and found the order of fluxes to be VMD > DCMD > AGMD. In fact, DCMD flux at the lab-scale is four times greater than that obtained for AGMD. However, at the pilot scale, the behavior was reversed and AGMD showed both higher flux and better energy efficiency compared to DCMD [57]. This reiterates the importance of complementing the development of new MD configurations with an investigation into the effects of upscaling these configurations, through both modeling and experiments, as the behavior may differ greatly between lab and pilot scale systems. In this review, we focus on various MD configurations that have evolved from the four conventional configurations discussed above along with novel configurations aimed at increasing the energy efficiency of MD systems.

## 2. Recent Developments in MD Configurations

Two aspects are considered when aiming to overcome challenges associated with MD today: membrane design and module configuration. Progress in MD membrane materials, as suggested earlier, is not of much use without accompanying focus on module and configuration design. Most improved and novel configurations seek to enhance thermal energy efficiency and vapor flux. New configurations stem from modification of AGMD and VMD, Some examples of modified MD configurations include material gap membrane distillation (MGMD) [71], vacuumed air gap membrane distillation (VAGMED), or sub-atmospheric AGMD [72,73] submerged membrane distillation (SMD) [74,75], conductive gap membrane distillation (CGMD), permeate gap or liquid gap membrane distillation (PGMD or LGMD) [76], flashed-feed-VMD [77], vacuum-enhanced DCMD [78] and vacuum multi-effect membrane distillation (V-MEMD) [79,80,81], (Hassan et al., 2020), [82]. Among the aforementioned MD configurations, V-MEMD and PGMD/LGMD have already been introduced into the market in the pilot scale module. V-MEMD is a kind of VMD configuration in which vacuum has applied in multiple stages and effects as in the case of conventional multi-effect distillation (MED) process. This section presents an overview of each of these new MD configurations.

### 2.1. Vacuum-Multi Effect Membrane Distillation (V-MEMD)

**Figure 3:**
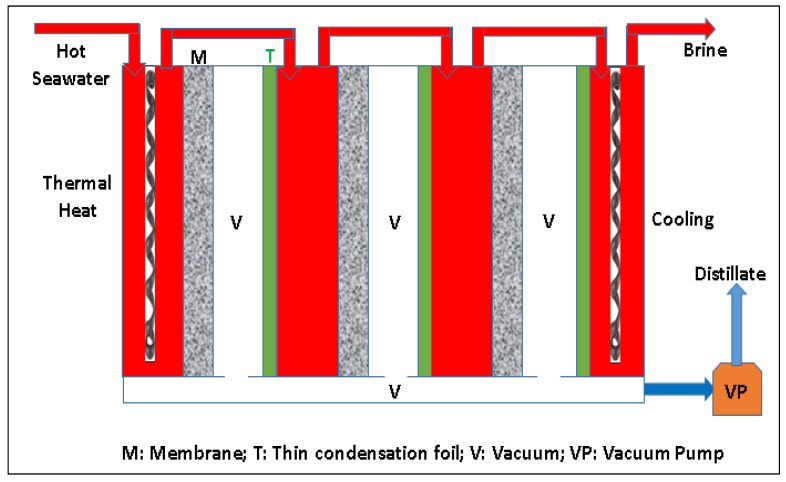
Schematic of the Vacuum Multi-Effect Membrane Distillation.

Multiple recycling of energy takes place in the V-MEMD system. A Teflon microporous membrane has been employed inside the plastic module between water space and vapor space. Hot feed solution is circulated through the water space and partial vapor pressure difference drives the permeate vapors from the feed solution side to the vapor space through the membrane pores. Feed solution in the following stage is separated from the vapor space through a thin polypropylene foil. Feed solution in the water space will be heated by the vapors in the foil frame, and then condensed. The heat energy of condensation is transported through the foil and converted to the evaporation energy of the feed, which generates new vapor in the vapor channel. Vacuum is applied in each vapor space and distillate can be collected outside the membrane module. Figure 3 shows the schematic representation of the V-MEMD process.

Condensed permeate will pass into a distillate channel. Pilot modules of V-MEMD configuration have been developed by MEMSYS and employed in different production capacities in many countries. According to Zhao and co-workers [81], the Memsys V-MEMD system is compact and highly energy-efficient to produce high-quality water using solar energy or waste heat sources. They studied the performance of 2-stage and 4-stage systems. GOR of 2-stage and 4-stage systems are found to be 1.84 and 2.79, respectively. They also studied the performance of 2-stage systems with 7, 9, and 17 frames and found that decreasing the flux with the increase in the number of frames, but the GOR is similar in all three cases. In 2016, New Concepts Holdings Limited (NCHL) acquired Memsys. They introduced standard models with various capacities from 3 to 24 tons/day distillate production depending upon the feed water types and qualities. Apart from the standard models, they are also capable of producing customized systems with a distillate production capacity of 50–1000 tons/day.

Andrés-Mañas and co-workers demonstrated the V-MEMD Memsys system as an innovative technology for the Mediterranean Seawater desalination [83]. They also observed enhanced heat recovery by preheating the seawater in the condenser. Wenzel and co-workers demonstrated a continuous 365-day long-term experiment using the V-MEMD Memsys module with a highly concentrated feed solution [84]. Feedwater concentration was about 9.5–22 wt. % and the distillate conductivity was measured to be less than 10 µS/cm throughout the experiment. This shows the reliability of the MD process and the capability of treating brines with high concentrations towards the zero liquid discharge (ZLD).

### 2.2. Material Gap Membrane Distillation (MGMD)

**Figure 4:**
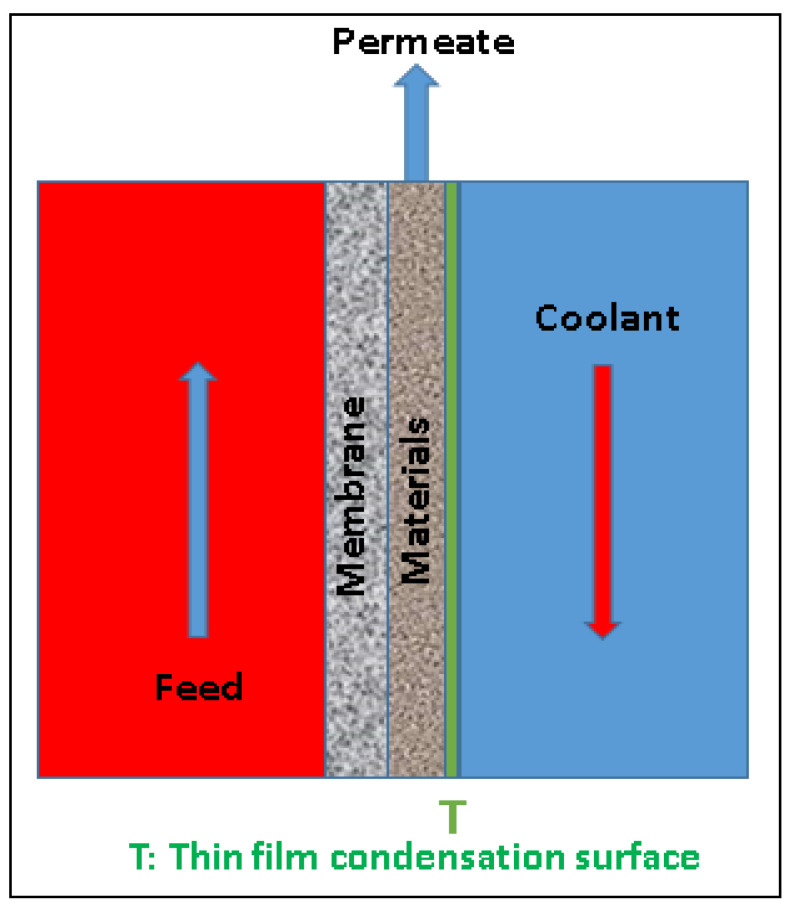
Schematic of a material gap membrane distillation (MGMD) module.

Francis and co-workers introduced the MGMD process [71]. It is a novel MD configuration in which different types of materials are filled in the air gap of an AGMD module. The air gap in an AGMD module causes a huge resistance for the mass transfer. Certain materials that filled in the air gap could reduce the mass transfer resistance and enhance the condensation process, therefore increasing the permeate flux. Figure 4 shows the schematic of the MGMD module. The materials used in the MGMD configuration are sand, polyurethane, conducting materials, water, etc. While using conducting materials, the MD module or the process is known as conductive gap membrane distillation (CGMD). On the other hand, many researchers have used water or permeate in the air gap of an AGMD module and name the process as water gap, liquid gap, or permeate gap membrane distillation (WGMD, LGMD, or PGMD). Francis et al. observed an increase in the water vapor flux of roughly 200–800% by filling the air gap of an AGMD module with different materials at various feedwater temperatures. The materials used in this study are sand, polypropylene, polyurethane and water in varying thicknesses. It was observed that the conducting materials can enhance the permeate flux whereas, the insulating materials such as polypropylene and polyurethane have no significant influence on the permeate flux. This is because of the occurrence of heat transfer hindrance which dominates over the air gap reduction. Apart from employing the different materials in the air gap, the influence of various hot feed/coolant velocities, various feed/coolant temperatures, and different gap widths are investigated and reported in this article. Figure 4 shows the schematic representation of the MGMD module.

Cai et al. in 2020, studied the transport analysis of the MGMD process and confirmed that the highly thermally conductive materials filled in the air gap of an AGMD module can significantly enhance the permeate flux [85]. As a follow-up to MGMD investigations, another research group from the Massachusetts Institute of Technology, USA extended this work by filling the conductive metallic meshes in the air gap of an AGMD module and compared with the PGMD process. They found that PGMD has a 20% higher gain-output ratio (GOR) or energy efficiency than that of an AGMD system of the same size and CGMD can have double the GOR than that of a PGMD system at similar operating conditions. This is due to the better energy recovery in a CGMD process into the cold stream within the MD module. The same research group has secured a US patent on an apparatus for energy-efficient conductive gap membrane distillation [86]. This patent claims the benefits and advantages of CGMD over AGMD and PGMD. Mahmoudi and co-workers have demonstrated a unique PGMD system for simultaneous production of power and freshwater [87]. It is demonstrated that at a ΔT of 65 °C, an optimum permeate hydraulic pressure of 1.1 bar, 12.5 LMH permeate flux and, 0.4 W/m^2^ of power density were achieved. Pelin and co-workers studied the computational fluid dynamics (CFD) modeling for the performance assessment of the PGMD process [88]. The developed model was validated using experimental data. It reveals the influence of module design parameters in maximizing the permeate flux and efficiency. With the help of theoretical and experimental studies, Baek-Gyu lm et al. recently reported, comprehensive insights into the performance of AGMD and WGMD processes using hollowfiber membranes [89]. They also predicted the minimum specific energy consumption (SEC) by optimizing the ratio of different hollowfiber membranes to the hollowfiber condensers.

### 2.3. Vacuumed AGMD and DCMD

**Figure 5:**
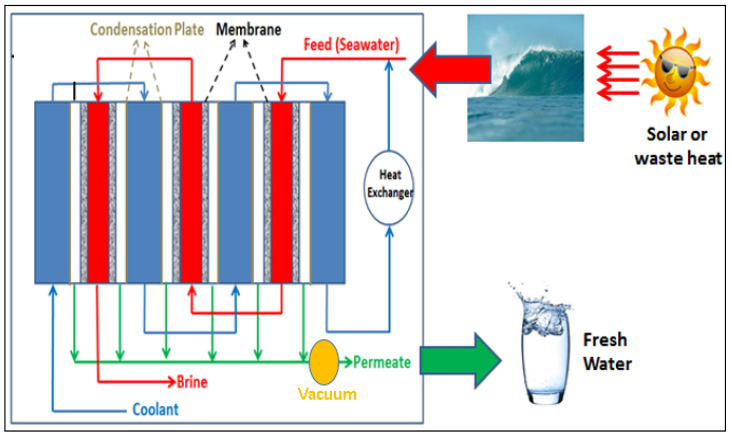
Schematic of a multi-stage VAGMED process.

AGMD process operated at sub-atmospheric conditions or under the application of controlled vacuum in the air gap of an AGMD module is known as vacuumed air gap membrane distillation (VAGMED) process, as reported by Ghaffour and co-workers [73]. As per the simulation and experimental findings of this report, the removal of non-condensable gases from the air gap of an AGMD enhances the permeate flux by three times when the gap is maintained at the saturation pressure of the feed temperature. In a multi-stage process operation, the feed temperature decreases from the first stage to the last stage due to the respective mass transfer and heat transfer, and the vacuum in the air gap of the module can be adjusted as per the saturation pressure of the feed solution. This is the difference between V-MEMD and VAGMED processes. They have also discussed the importance and enhanced efficiency of the MD process that could be achieved by proper engineering and efficient staging of the process. Membrane pore wetting is less likely in this system compared to conventional VMD. A schematic of a multi-stage VAGMED process is shown in Figure 5. The effect of staging on the permeate flux in the VAGMED system and its relation to the membrane cost is also briefly discussed in this study. In this process, it is important to keep in mind that the more the gap pressure is reduced below the saturation pressure of the feed temperature, the lower the cooling temperature required for permeate condensation. Therefore, it is recommended to keep the gap pressure slightly below the saturation pressure of the feed temperature for the complete removal of the non-condensable gases from the air gap and to overcome the mass transfer resistance in the membrane structure. Thus, VAGMED is a combination of AGMD and VMD processes with the aid of controlled engineering measures for multi-staging of the process which enhances the efficiency and water production. Recently, Kim and co-workers investigated powdered activated carbon (PAC)—vacuum-assisted AGMD (V-AGMD) hybrid system to treat the wastewater containing surfactants. They found that PAC removes the surfactants in the wastewater via adsorption and mitigates membrane wetting in the MD process. They also mentioned that the vacuum pressure should be less than the liquid entry pressure of the membrane to prevent wetting [90].

Okiel et al. investigated the vacuum-enhanced DCMD (VE-DCMD) process for oil field-produced water desalination [78]. They derived the SEC and energy efficiency of the process along with the performance comparison of pristine and modified polypropylene (PP) membranes. Naidu and co-workers demonstrated a novel vacuum-enhanced DCMD (V-DCMD) process in which they investigated the transport phenomena and fouling behavior using experimental and theoretical modeling. V-DCMD enhanced the permeate flux by 37.6% compared to the DCMD configuration [91]. This is because of the removal of non-condensable gases within the membrane pores upon the application of vacuum. Fahmey et al. demonstrated a scale-up approach through V-DCMD for water desalination [92]. They also studied the comparative performance evaluations of nanomaterials mixed polysulfone membranes.

### 2.4. Submerged Membrane Distillation (SMD)

**Figure 6:**
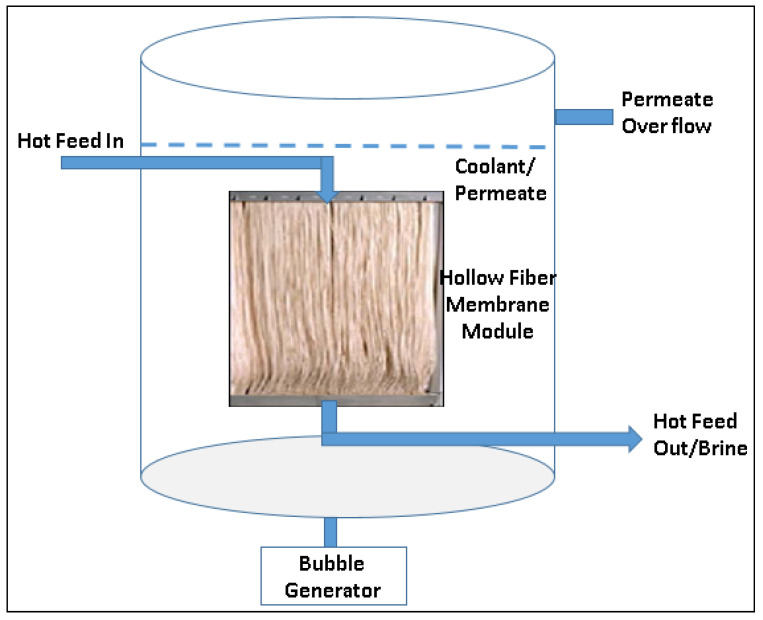
Schematic of Submerged membrane distillation (SMD) process.

SMD is a recently introduced MD process configuration. In the SMD process membrane module is submerged either in a feed solution tank or in a coolant stream. Module design and construction are very simple in an SMD configuration compared to other MD configurations. Apart from module simplicity, a major advantage of SMD is that it can be easily employed in other conventional MD configuration modes such as DCMD, VMD, and SGMD. Francis and co-workers [74] have carried out lab-scale tests on SMD process using hollow fiber membranes and reported that the permeate flux is comparable with the other conventional MD configurations. Figure 6 shows a schematic of SMD process configuration using a hollow fiber membrane module. A flat sheet membrane with a plate-and-frame closed module can also be employed in the SMD process. Figure 6 shows the SMD process in DCMD mode in which an open membrane module is submerged in the cold stream and a hot feed stream flows through the lumen side of the membrane. It is also possible to design in the other way such as the membrane module can be submerged in the hot feed stream and coolant stream can be passed through the lumen side of the membrane. One or more bubble generators are be used to reduce the temperature polarization phenomenon during the operation. Researchers have also been reported the use of microbubbles in the feed solution to enhance the permeate flux [93]. In a submerged VMD process, a hot feed stream can be passed through the lumen side of the hollow fiber membrane bundle and a vacuum could be applied at the shell side of the membrane bundle fixed in a membrane module or vice versa. This work has been published as a US patent application in 2016 [75].

Julian and coworkers have reported an integrated submerged vacuum membrane distillation crystallization (SCMDC) system for the water recovery and cane sugar crystallization. [94] They observed extremely low permeate flux due to the high degree of impurities in the feed solution. Choi and co-workers have reported an integrated submerged membrane distillation—adsorption system for simultaneous water reclamation and Rubidium recovery. This work is demonstrated as a model for the extraction of valuable resources from the feed solution via the MD process integrated with the adsorption method. Potassium copper hexacyanoferrate is used as the adsorbent [95]. Meng et al. investigated submerged VMD systems for inland desalination applications [96]. They compared submerged VMD systems with conventional MD configurations. An increase in the permeate flux was observed with the application of transverse vibration. Periodical aeration and air backwash could help to mitigate scaling issues with complex feeds. Choi et al. have studied a fractional–submerged membrane distillation crystallizer (F-SMDC) for the treatment of highly saline water [97]. They found that F-SMDC is effective in the value-added crystallization process and produces a high-quality distillate with fewer membrane scaling problems. In another context, Gryta M also studied the application of submerged modules for the MD process. Higher values of permeate flux were obtained when the membranes were immersed in the feed with the distillate flowing inside the capillary membranes. The process efficiency of the submerged MD process was additionally compared with the conventional MD capillary modules and similar performance of both configurations was achieved [98]. Bae W and Kim J recently investigated on submerged DCMD process for the treatment of synthetic wastewater [99]. Permeate flux was found to be increased with the increase in the feed mixing intensity and recirculation velocity of the permeate at the lumen side of the polyethylene hollow fiber membranes. Recently, Bamasag et al. investigated a solar-heated submerged -VMD system with agitation techniques [100]. The hollow fiber membrane module was submerged in an evacuated solar tube filled with a feed water solution. Aeration and internal circulation inside the feed solution were used as the agitation techniques, which helped in reducing the TP effect, and as a result, permeate flux enhanced up to 22%. Overall, permeate flux is not very high but SMD module design is the simplest design ever discussed in MD module configurations and this design could reduce the overall life cycle cost of the process in large-scale production.

### 2.5. Flashed-Feed VMD (FF-VMD)

**Figure 7:**
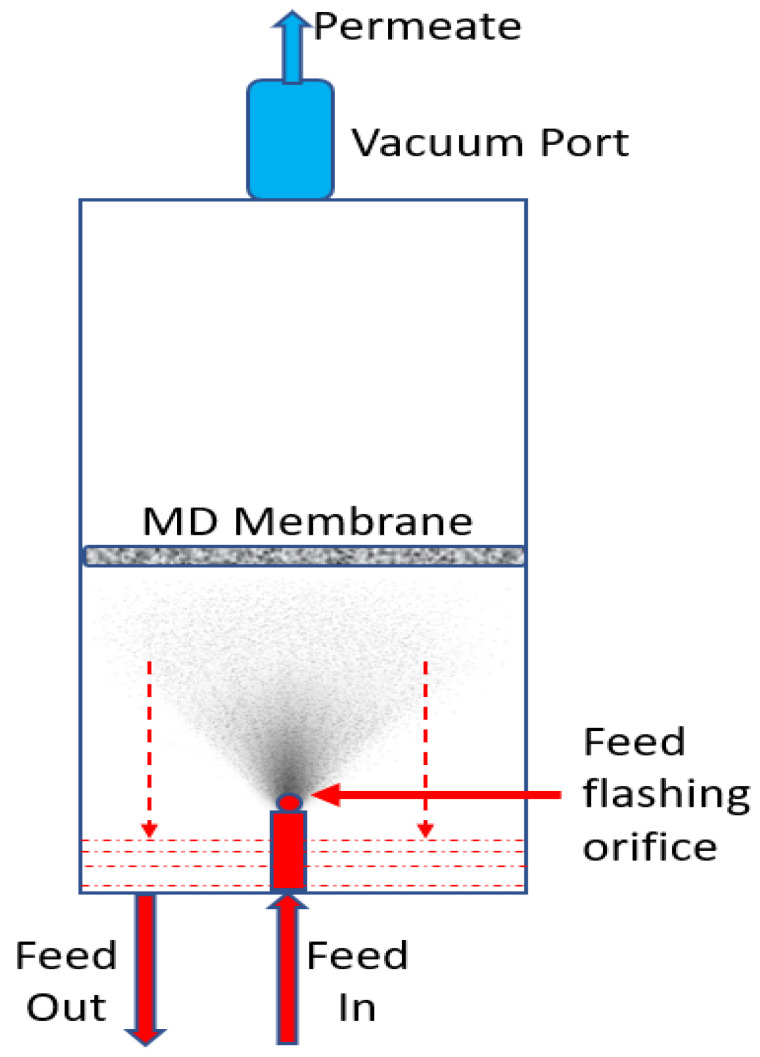
A schematic of the FF-VMD membrane module.

Al Saadi and co-workers introduced another interesting and improvised VMD design which is known as Flashed-feed VMD (FF-VMD) configuration [77]. Flashed-feed VMD configuration as a novel method for eliminating temperature polarization effect and enhancing water vapor flux. In FF-VMD, the feed solution is not in direct contact with the membrane but flashed through a very small orifice into the feed chamber to mitigate the TP effect. As a result, flashed-feed VMD configuration yields enhanced permeate flux as high as 3.5-fold (200 LMH) higher than the conventional VMD process under similar operating conditions. A very simple schematic of the FF-VMD membrane module is represented in Figure 7. In this investigation, the TP effect was decoupled from the membrane mass transfer coefficient by avoiding the hot feed stream from directly contacting the membrane surface. It is concluded from this study that the heat transfer coefficient controls the resistance of permeate flux in a conventional VMD configuration. FF-VMD reports permeate flux values as high as 9 LMH and 40 LMH at ΔT of 5 °C and 10 °C, respectively, at a feed temperature of 70 °C.

### 2.6. Dead-End Membrane Distillation (DE-MD)

**Figure 8:**
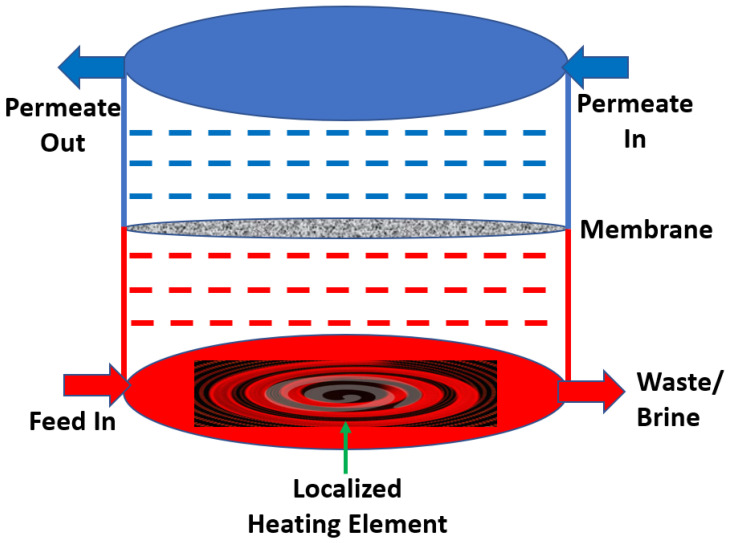
Schematic representation of DE-MD Module.

Mustakeem et al. recently introduced a novel dead-end MD (DE-MD) module configuration with localized interfacial heating for sustainable and energy-efficient desalination [101]. In this system, heating of the feed solution is taking place inside the membrane module with the help of a localized heating element installed at the feed chamber. Intermittent flushing of the feed solution could reduce the TP effect to enhance the permeate flux. Specific energy consumption decreased up to 57%, permeate flux increased up to 45% and GOR value increased up to 132 ± 12% with this novel MD module configuration. A schematic diagram of the DE-MD module is represented in Figure 8. Implementation of DE-MD could also improve the membrane fouling issues and conventional heat losses in the MD process. Conjugate heat transfer model studies reveal that localized heating technique provides a uniform heat transfer across the membrane and the process becomes more efficient.

In fact, localized heating at the membrane surface using electrothermal heating has recently been proposed to improve process efficiency via suppression of the thermal boundary layer. Dudchenko et al. were the first to apply localized Joule heating using carbon nanotubes [102]. They demonstrated an increase in GOR as the heated membrane surface reduces effects of TP. Since then, various groups have applied Joule heating to DCMD [103,104], AGMD [105] and VMD [106]. In VMD, the application of an electrothermal braid-reinforced PVDF hollow fiber membrane showed 2.5 times flux enhancement and significant suppression of TP. In another study, [107], Subrahmanya et al. applied a graphene-PVDF flat sheet membrane to VMD for seawater desalination and achieved a permeate flux of 23.44 L m-2 h-1 at a low specific heating energy consumption of 0.109 kWh L^−1^.

**Figure 9:**
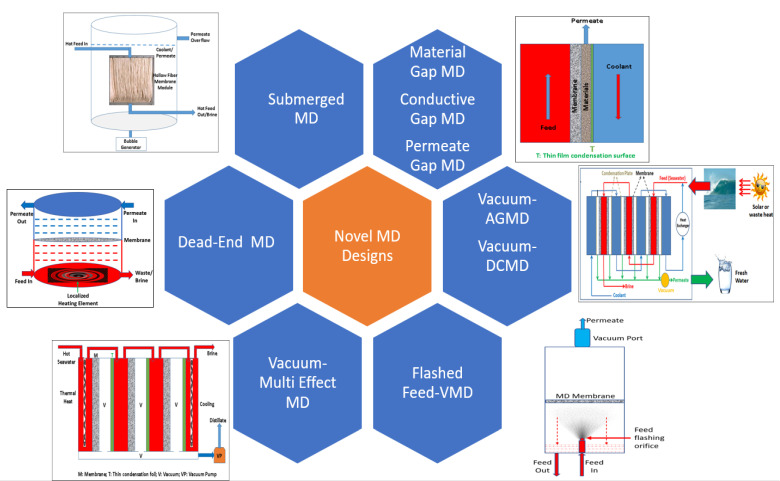
Novel MD module designs.

All the above-described novel MD module configurations are summarized in Figure 9.

## 3. Developments in Pilot Scale MD Technologies

Scarab Development AB (Stockholm, Sweden), Fraunhofer ISE- Solar Springs GmbH (Freiburg, Germany), TNO—Memstill (Amsterdam, The Netherlands), Aquastill (Sittard, The Netherlands), Aquatech (Hackettstown, NJ, USA), Memsys GmbH (Schwabmünchen, Germany), KmX Corporation (Markham, ON, Canada), Memsift Innovations (Pandan Loop, Singapore) and Econity (Gyeonggi-do, Korea) are some commercial MD pilot technology developers. Scarab developed an AGMD membrane module whereas, Solar springs, Memstill, and Aquastill developed LGMD/PGMD membrane modules in spiral-wound design using flat sheet membranes. However, it is very important to mention that several MD pilot plants integrated with renewable solar energy have been successfully installed and operated, recently. Table 2 shows the different MD pilot systems installed and operated with different MD configurations and production capacities.

Solar Spring GmbH was founded in 2009, which is a spin-off of the Fraunhofer Institute for Solar Energy Systems in Freiberg, Germany. Winter et al. [110] developed spiral-wound AGMD and PGMD modules for solar-driven MD desalination applications.

Memsys water technologies GmbH from Germany, use their V-MEMD configuration with PTFE membrane evaporators and thin-film PP condensers aligned in a plate and frame model. Memsys technology uses V-MEMD process design in all their systems. They claim freshwater production with enhanced energy recovery and efficiency. The technical description of V-MEMD is described in Section 2.1.

Aquastill was founded in 2008 and they are interested in MD technology with a unique design, which was developed in the Netherlands. It has an AGMD spiral-wound module with a low-density polyethylene membrane. Aquastill MD pilot plants have been installed and tested at many universities in the USA, Europe, Australia, South Korea, the Middle East, and Africa, with a production capacity of 3–10 m^3^/day.

Scarab developments AB is a Swedish company, founded in 1973. They patented the plate-and-frame AGMD module design in 1981. flat sheet modules. The Swedish company Xzero secured the license from Scarab to use their technology in the semiconductor industry [119]. PTFE MD membranes in Scarab modules have a nominal porosity of 80%, a thickness of 0.2 mm, and an average pore size of 0.2 µm. Each module contains 10 plastic cassettes with two membranes each and a total membrane area of 2.3 m^2^ [115]. Zaragoza et al. [116] investigated different MD pilot systems from various manufacturers such as Scarab AB, Keppel Seghers, Solar Spring, and Aquaver-Memsys. As mentioned above, Scarab AB provides flat sheet AGMD module, Keppel Seghers provides flat sheet LGMD, Solar Spring provides spiral-wound LGMD and Aquaver-Memsys has V-MEMD modules. They coupled solar thermal energy with MD pilot systems and concluded their study that spiral-wound modules or multi-effect MD modules should be considered for better heat recovery and energy efficiency.

Aquatech is a US-based company that provides advanced VMD (AVMD) MD modules for zero liquid discharge (ZLD) and minimum liquid discharge (MLD) facilities. Despite the conventional MD configurations, the flat sheet membrane acts only as a demister and it does not touch the feed solution. They filed the patent application for the “method and apparatus for the advanced vacuum membrane distillation” [120].

US-based KMX Technologies LLC and Singapore-based Memsift Innovations are recently introduced hollow fiber membrane-based MD pilot modules. Hollow fiber membranes can provide low footprint-large surface area modules. Memsift MD process uses a Carnote cycle based on the Joule-Thomson effect to control the temperature. KMX technology is based on PTFE hollow fiber membranes with VMD configuration for Lithium recovery, acid mine drainage, and produced water treatment [82].

Jia et al. [121] developed PTFE hollow fiber membrane-based VMD module with an 8.2 m^2^ selective membrane area. They investigated eight similar hollow fiber membrane modules in a system to have a total membrane area of 65.6 m^2^ to produce 8 m^3^ per day fresh water to treat simulated wastewater in a nuclear power plant. They were able to achieve a high decontamination factor and concentration factor. They also analyzed the heat loss of a pilot scale membrane module as a heat pinch effect.

## 4. Niche Applications of MD

**Figure 10:**
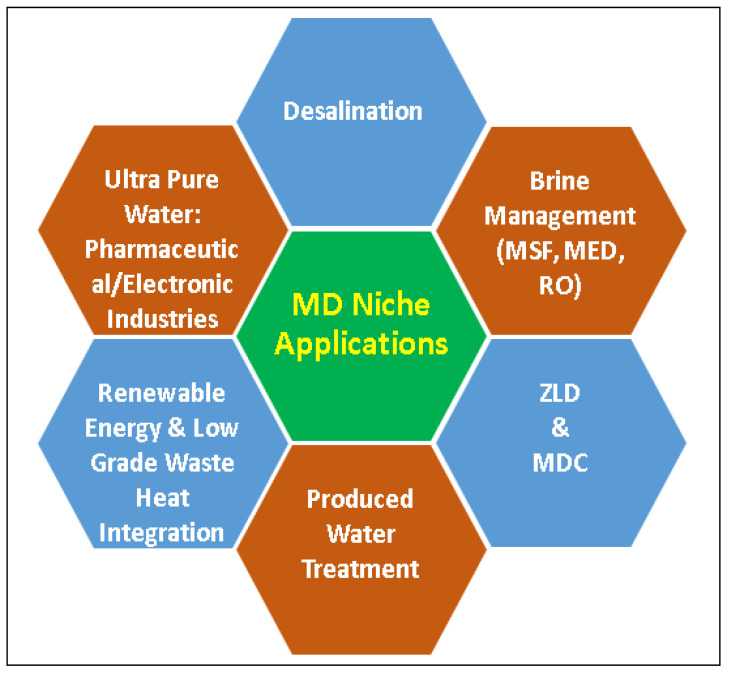
Niche applications of MD process.

Apart from seawater desalination, there are several applications where MD process can be used in the reclamation of freshwater. Brine management or brine treatment is one among them in which the MD process can be integrated with conventional desalination plants such as MSF, MED, and RO, and use brine from these plants to extract freshwater. Since MD can operate at very high salinity, the industry could avail the benefit of the MD process to reduce the brine volume and reduce the difficulties in brine disposal. MD can even be applied for zero liquid discharge (ZLD) or minimum liquid discharge (MLD) process and recover the valuable minerals in the brine using a process known as membrane distillation crystallization (MDC). A huge volume of wastewater is generated every day in the oil and gas industry. Treating this produced water is a possible niche application of the MD process. MD process could reject almost 100% salts and nonvolatile contaminants and produce ultrapure water for pharmaceutical and electronic industries. Niche applications of the MD process can be demonstrated as shown in Figure 10 [122,123].

## 5. Energy Efficiency of MD Configurations

The thermodynamic limit for desalinating seawater with a salinity of 3.5% and at the rate of 50% water recovery is calculated as 1.06 kW h m^−3^ [123]. Since phase change occurs during MD operation, it can be economically viable only with the availability of low-grade energy sources. The energy efficiency of various MD processes reported in the literature is highly variable as there is no standardized methodology for the energetics and cost calculation for MD. This wide range of energy values is due to the various operating conditions, different feed water qualities and module size. Operating expenses for any MD process configurations are more or less similar. Engineered membranes and engineered processes can have a significant impact on the energy efficiency of the MD process. Highly porous, robust, and wetting/fouling resistant membranes are considered to be the most efficient membranes for the MD process, whereas properly engineered processes can play a very important role in minimizing heat loss and mitigating TP. Recently developed innovative MD configurations consider latent heat recovery and mitigation of TP phenomenon to enhance energy efficiency. Najib et al. studied the energy and exergy efficiency of the V-MEMD system [124]. They calculated the specific thermal energy consumption (STEC) and specific electrical energy consumption (SEEC) of the V-MEMD system as 166 kWh/m^3^ and 4.5 kWh/m^3^, respectively. Under ideal operational conditions, the exergetic efficiency reached 21.1%. They also observed that the maximum fraction of exergy destruction was localized in the condensation compartment. In another report, Ullah et al. [58] found that the required thermal energy and electrical energy of the DCMD process is 100 kWh/m^3^ and 1.5–3.65 kWh/m^3^, respectively for a recovery of 60–80%. Chang-Kyu Lee et al. conducted an experimental study of AGMD (Aquastill modules) pilot with a production capacity of 10 m^3^ per day. During the 50-day operation of this pilot plant, they evaluated the STEC for tap water, seawater, and concentrated brine as 182–208 kWh/m^3^, 199–231 kWh/m^3^ and 294–373 kWh/m^3^, respectively at various operating conditions [125]. Christie et al. [126] developed a framework for evaluating and optimizing the energy efficiency of the DCMD process. As per their findings, multi-staging the DCMD process by employing heat exchangers in each stage of the process could enhance the efficiency and GOR. They also elucidated a new metric, namely specific yield to quantify the performance of DCMD powered by waste heat stream.

As discussed earlier, in a comparative study of AGMD, PGMD, and CGMD, PGMD showed 20% higher GOR and CGMD showed two times the GOR than that of PGMD configuration [76]. The enhancement in GOR indicates enhancement in energy efficiency. This is because of the possibility of intra-modular energy recovery during the process while using conductive spacers in the air gap of an AGMD module. Okiel et al. studied the SEC and energy efficiency of the VE-DCMD process for oil field-produced water desalination. According to their investigation, desalinating oil field water with feed salinity of 10,000 ppm and 230,000 ppm using the VE-DCMD system requires 3176.4 kWh/m^3^ and 4189.5 kWh/m^3^, respectively [78]. Andrés-Mañas and co-workers demonstrated V-AGMD system using spiral-wound modules with low STEC of 49 kWh_th_/m^3^ with a GOR of 13.5. Removal of non-condensable gases from the air gap of an AGMD system using a vacuum is the reason for enhanced permeate production and efficiency. They also observed that hypersaline brines can be treated with STEC lower than 515 kWh_th_/m^3^ and GOR of 1.2. Chang et al. investigated the performance and efficiency analysis of a submerged VMD system, numerically [114]. They evaluated the desalination performance and energy efficiency through simulation and validated via experiments. In this study, the predicted GOR could be achieved by combining higher feed temperature and higher feed flow rates. It is important to note that higher or optimum packing density can significantly impact the performance of the S-VMD system.

In a recent study, Alsaati and Marconnet observed that locally heated MD systems can reduce the energy consumption up to 75% compared to the conventional MD systems at higher temperatures [127]. They used self-assembled monolayer coated thermally stable silver membranes and other commercially available MD membranes for this study. DE-MD system promotes localized heating with the aid of heating elements inside the membrane module. This system addresses the TP issues and reduces heat loss throughout the process. As per the observation of the authors in this work, mitigation of TP increases the trans-membrane vapor pressure difference which causes a 10–45% increase in the permeate flux, 44–57% reduction in the SEC and, a 132% increase in GOR. DE-MD systems can achieve SEC up to 1183 kWh/m^3^ which is approaching the thermodynamic energy limit for water evaporation (650 kWh/m^3^) (101). FF-VMD system also tried to address the problems of the TP phenomenon—which could reduce the bulk feed temperature up to 10 °C. Flashing the feed by not being in direct contact with the membrane surface minimizes TP and enhances permeate flux even at very low ΔTs. Membrane lifetime is maximized compared to other configurations due to low propensity of wetting and fouling in FF-VMD [77].

Criscuoli recently studied the thermal performance of an integrated DCMD-VMD system and observed a 50% reduction in the STEC, 100% increase in GOR, and 69% increase in the permeate production as compared to DCMD alone. Importantly, the analysis was carried out at very low operating temperatures (~40 °C) so that low-grade waste heat sources can be effectively used [128]. Cost and excess energy required for heating elements, use of conductive materials, excess vacuum, localized heating, and flashing of the feed have to be included in the energy efficiency analysis. This is not clear from reported literature. Furthermore, as mentioned above, direct comparison of various configurations is extremely difficult due to non-standardization. More studies on the direct comparison of different configurations for the same feed and operating conditions and production capacity must be carried out.

## 6. Conclusions

As an emerging and attractive water reclamation process, global interest in membrane distillation process design is growing, especially for desalination and water treatment. Certain bottlenecks that hinder MD from large-scale commercialization include mitigation of TP, membrane design, and inefficient heat and mass transfer due to poor system design. While membrane design has been the focus of many studies, development of appropriate system configurations for optimal energy efficiency for each application has received considerable attention, and is a critical aspect in advancing MD. Lab-scale and pilot scale investigations have proved that the MD is a promising technology for niche applications such as brine treatment, recovery of valuable metals (ZLD/MLD), and ultra-pure water production for the semiconductor industry. MD plants can be installed as stand-alone modular units in remote/arid regions and they can be integrated with existing desalination plants to reuse the brine, reduce the waste, and recover freshwater. Reusing and reducing the volume of waste that was supposed to be disposed of has many positive environmental implications. Use of waste heat or renewable energies (solar, geothermal and waste heat from power plants) for the MD process operation are very important and MD technology can be thus called the renewable energy-driven water reclamation process. Degassing of the feed solution is important if it contains any volatile pollutants as the MD process cannot resist volatile impurities through the membranes. V-AGMD and V-MEMD processes are found to be more energy-efficient compared to other modules/configurations. Spiral-wound PGMD designs are also recommended for their compactness and energy/water recovery. Contactless feed solution (FF-VMD and Aquatech’s A-VMD) and localized heating (DE-MD) processes are recently developed MD technologies and much more to come in the recent future to enhance the permeate production and energy efficiency by mitigating the TP phenomenon. The cost of the heating elements and conductive materials used in the membrane modules, and the excess energy consumption required for these alterations needs to be taken account while calculating the energy matrixes. Hollow fiber-based pilot scale modules and designs are another area to explore the possibilities of energy and cost reduction in the MD process. A holistic life cycle cost analysis (LCCA) with the aid of standardized tools for the analysis of energy efficiency, STEC, SEEC, and GOR still needs to be developed to normalize the energy and cost metrics of MD process. Future direction in MD configurations also includes developing materials for surface heated MD and combining these with the configurations discussed.

## Figures and Tables

**Table 1:** Advantages and disadvantages of each of the four conventional membrane distillation configurations [68,69].

Configuration	Advantages	Disadvantages
DCMD	Ease of operation	High conductive heat loss
AGMD	High thermal efficiency due to presence of air gap	Low permeate flux Air gap increases mass and heat transfer resistance
SGMD	Sweeping gas improves mass transfer at permeate side Low conductive loss through membrane	Complex module design Difficult heat recovery
VMD	High permeate flux	Greater risk of membrane wetting

**Table 2:** MD pilot systems deployed in different parts of the world.

Company	Location (Year)	Configuration	Application
Memstill [108,109]	Singapore (2006–2007)	Flat Sheet AGMD	Polluted Seawater Desalination
	Netherlands (2006–2007)		Brackish Seawater Desalination
	Netherlands (2008)		Polluted Brackish Water
Fraunhofer Solar Spring GmbH [50,110]	Italy (2010)	Flat Sheet Spiral-Wound AGMD and PGMD	Waste Heat Driven Seawater Desalination (5 m^3^/day)
	Namibia (2011)		Solar Thermal Ground Water Desalination (5 m^3^/day)
	Spain (2011)		Solar Thermal Seawater Desalination (5 m^3^/day)
Memsys PTFE membrane [79,80,81,111]	Singapore (2012)	Plate and Frame V-MEMD	Solar and waste heat driven Seawater desalination (<1 m^3^/day)
	Qatar (2014)		Seawater and Thermal brines (<1 m^3^/day)
	Saudi Arabia (2015)		Four stage-single effect system optimized for 43–46 °C feed.
	Greece (2016)		Artificial Saline Water Desalination (30–50 LMH)
Aquastill Low-density polyethylene membrane [112,113,114]	Australia (2015)	Spiral-wound AGMD	7.2 m^2^ membrane area, >1 LMH. Seawater and synthetic seawater as feed. GOR up to 9
	Spain (2017)	Spiral-wound AGMD	Two pilot modules with membrane area 7.2 m^2^ and 24 m^2^. Seawater desalination application. 1.35–4.2 LMH
	Spain (2020)	Spiral-wound V-AGMD	Two pilot modules with membrane area 7.2 m^2^ and 25.9 m^2^. Seawater desalination application. 8.7 LMH, GOR 13.5. The longest module has maximum efficiency, but low flux.
Scarab AB-Xzero [115,116]	Sweden (2010)	Flat sheet Plate and Frame AGMD PTFE membrane	2.3 m^2^ membrane area, Municipal wastewater as feed. 35% recovery. Significant flux decay after 370-h continuous operation.
	Spain (2014)		2.8 m^2^ membrane area, synthetic brackish water, and seawater desalination application. 6.5 LMH
Econity—Global MVP [117,118]	South Korea (2016, 2017)	PVDF Hollow Fiber VMD module	4 LMH at 56 °C 35,000 ppm feed solution.
			5.3 m^2^ active membrane area. 18 LMH flux at 75 °C, with 99.99% rejection of inorganic salts.

## Data Availability

Not applicable.
